# Supplementary material for: Poincaré sphere trajectory encoding metasurfaces based on generalized Malus’ law
Source: Nat Commun. 2024 Mar 16;15:2380. doi: 10.1038/s41467-024-46758-y (PMC10944530; doi:10.1038/s41467-024-46758-y)
Supplement: Supplementary file 1 — Supplementary Information [file 41467_2024_46758_MOESM1_ESM.pdf]

Supplementary Information for
Poincaré Sphere Trajectory Encoding metasurfaces Based on Generalized Malus' Law

Zi-Lan Deng[1,*], Meng-Xia Hu[1], Shanfeng Qiu[2], Xianfeng Wu[2], Adam Overvig[3], Xiangping Li[1,*], Andrea Alù[3,,4,*]

[1]*Guangdong Provincial Key Laboratory of Optical Fiber Sensing and Communications, Institute of Photonics Technology, College of Physics & Optoelectronic Engineering, Jinan University, Guangzhou 510632, China.*

[2]*Shphotonics LLC, Suzhou, 215000, China*

[3]*Photonics Initiative, Advanced Science Research Center, City University of New York, New York, NY 10031, USA*

[4]*Physics Program, Graduate Center, City University of New York, New York, NY 10016, USA*

[*]E-mail: zilandeng@jnu.edu.cn, xiangpingli@jnu.edu.cn, aalu@gc.cuny.edu

CONTENTS

1. Detailed derivation of the analytic formula of generalized Malus' law on the both the surface and the solid Poincare sphere........3

1.1. Generalized Malus' law on the surface Poincare sphere........3

1.2. Further extension of generalized Malus' law to the solid surface Poincaré sphere........7

2. Detailed design procedure for the Poincare sphere trajectory/grid encoding and decoding. 8

3. Explicit modulation relations between metasurface parameters and the generated polarizations covering the full Poincare sphere........11

4. Construct of the overall Poincare sphere analyzer with GML by cascading a 1/4 waveplate and a linear polarizer. 12

Supplementary Notes

1. Detailed derivation of the analytic formula of generalized Malus' law on the both the surface and the solid Poincare sphere

1.1. Generalized Malus' law on the surface Poincare sphere

To derive the generalized Malus' law (GML), let's first examine the full polarization representation on the surface Poincare sphere (PS) [1-8]. As shown in Supplementary Fig. 1, an arbitrary polarization state can be described by two PS parameters, namely, the azimuth ψ that describes orientation angle of the polarization ellipse, and the ellipticity χ whose magnitude describes the aspect ratio of the polarization ellipse and sign describes the helicity of the polarization. As illustrated in Supplementary Fig. 1 (a, b), the PS is defined as a unit-sphere, the PS parameters relate to the spherical coordinates (r; φ, θ) as

$$r=1,\ \varphi=2\psi,\ \theta=\pi/2-2\chi. \tag{S1}$$

A polarization state can also be described by the Stokes parameters (S_1, S_2, S_3), which are equivalent to Cartesian coordinates (x, y, z) of the PS as,

$$x=S_1,\quad y=S_2,\quad z=S_3. \tag{S2}$$

The relationships between the PS parameters and Stokes parameters can be derived by the transformation between Cartesian coordinate and sphere coordinate as follows,

$$\begin{pmatrix} x \\ y \\ z \end{pmatrix}=\begin{pmatrix} \sin\theta\cos\varphi \\ \sin\theta\sin\varphi \\ \cos\theta \end{pmatrix} \quad => \quad \begin{pmatrix} S_1 \\ S_2 \\ S_3 \end{pmatrix}=\begin{pmatrix} \cos 2\chi\cos 2\psi \\ \cos 2\chi\sin 2\psi \\ \sin 2\chi \end{pmatrix}, \tag{S3}$$

$$\begin{pmatrix} r \\ \varphi \\ \theta \end{pmatrix}=\begin{pmatrix} \sqrt{x^2+y^2+z^2} \\ \arctan\left(y/x\right) \\ \arctan\left(z/\sqrt{x^2+y^2}\right) \end{pmatrix} \quad => \quad \begin{pmatrix} 1 \\ \psi \\ \chi \end{pmatrix}=\begin{pmatrix} \sqrt{S_1^2+S_2^2+S_3^2} \\ \frac{1}{2}\arctan\left(S_2/S_1\right) \\ \frac{1}{2}\arctan\left(S_3/\sqrt{S_1^2+S_2^2}\right) \end{pmatrix}. \tag{S4}$$

Therefore, once the azimuth and ellipticity parameters (ψ, χ) are known, the Stokes parameters S_1, S_2, *and* S_3 can be explicitly obtained by Eq. (S3). On the other hand, once the Stokes parameters S_1, S_2, *and S3* are given, the azimuthal and ellipticity parameters (ψ, χ) can be explicitly obtained by Eq. (S4).

At the linear polarization basis $(\hat{\mathbf{x}},\hat{\mathbf{y}})$. A polarization state with PS parameters (ψ, χ) is represented by the following Jones vector [9, 10],

$$\boldsymbol{\alpha}(2\psi,2\chi)=\begin{pmatrix} \alpha_x \\ \alpha_y \end{pmatrix}=A_0\exp\left(i\Phi\right)\begin{pmatrix} \cos\psi & -\sin\psi \\ \sin\psi & \cos\psi \end{pmatrix}\begin{pmatrix} \cos\chi \\ -i\sin\chi \end{pmatrix}, \tag{S5}$$

where A_0 and Φ are the overall amplitude and phase retardation, which do not affect the polarization state and thus are ignored in the following discussion.

According to Eq. (S5), the right handed polarization (RCP) (coordinate: $2\psi=0^o$, $2\chi=90^o$) and left handed polarization (LCP) (coordinate: $2\psi=0^o$, $2\chi=-90^o$), which are located at the south and north

poles of the PS, respectively, could be written in the linear basis as follows,

$$\boldsymbol{\alpha}_{RCP}=\frac{\sqrt{2}}{2}\begin{pmatrix}1\\-i\end{pmatrix},\ \ \boldsymbol{\alpha}_{LCP}=\frac{\sqrt{2}}{2}\begin{pmatrix}1\\i\end{pmatrix}, \tag{S6}$$

Then, the base transformation matrix between linear and circular bases is [11, 12]

$$\boldsymbol{\Lambda}=\left(\boldsymbol{\alpha}_{RCP},\boldsymbol{\alpha}_{LCP}\right)=\frac{\sqrt{2}}{2}\begin{pmatrix}1 & 1\\-i & i\end{pmatrix}, \tag{S7}$$

while its inverse matrix is

$$\boldsymbol{\Lambda}^{-1}=\left(\boldsymbol{\Lambda}^{T}\right)^{*}=\frac{\sqrt{2}}{2}\begin{pmatrix}1 & i\\1 & -i\end{pmatrix}, \tag{S8}$$

Therefore, under the circular polarization basis $\left(\hat{\boldsymbol{\alpha}}_{RCP},\ \hat{\boldsymbol{\alpha}}_{LCP}\right)$, an arbitrary polarization state with PS parameters (ψ, χ) can be written as

$$\begin{aligned}\boldsymbol{\alpha}^{C}=\begin{pmatrix}\alpha_{RCP}\\\alpha_{LCP}\end{pmatrix}&=\boldsymbol{\Lambda}^{-1}\boldsymbol{\alpha}=\frac{\sqrt{2}}{2}A_0\exp\left(i\Phi\right)\begin{pmatrix}1 & i\\1 & -i\end{pmatrix}\begin{pmatrix}\cos\psi & -\sin\psi\\\sin\psi & \cos\psi\end{pmatrix}\begin{pmatrix}\cos\chi\\-i\sin\chi\end{pmatrix}\\&=\frac{\sqrt{2}}{2}A_0\exp\left(i\Phi\right)\begin{pmatrix}e^{i\psi} & ie^{i\psi}\\e^{-i\psi} & -ie^{-i\psi}\end{pmatrix}\begin{pmatrix}\cos\chi\\-i\sin\chi\end{pmatrix}\\&=\frac{\sqrt{2}}{2}A_0\exp\left(i\Phi\right)\begin{pmatrix}\left(\cos\chi+\sin\chi\right)e^{i\psi}\\\left(\cos\chi-\sin\chi\right)e^{-i\psi}\end{pmatrix}\\&=A_0\exp\left(i\Phi\right)\begin{pmatrix}\cos\left(\pi/4-\chi\right)e^{i\psi}\\\sin\left(\pi/4-\chi\right)e^{-i\psi}\end{pmatrix}\end{aligned}. \tag{S9}$$

As we can see, the circular polarization basis ensures that the amplitudes and phase are independently characterized by the azimuth and ellipticity parameters (ψ, χ) [13], as

$$A_{RCP}=\cos\left(\pi/4-\chi\right),\ \ A_{LCP}=\sin\left(\pi/4-\chi\right),\ \ \varphi_{RCP}=\psi\ ,\ \ \varphi_{LCP}=-\psi\ . \tag{S10}$$

Assuming that the polarized light with PS parameters (ψ, χ) passing through a circular polarizer that allows perfect transmission of RCP, while completely blocking its orthogonal state LCP, the transmitted intensity can be written as

$$I_{out}=A_0^2A_{RCP}^2=A_0^2\cos^2\left(\frac{\pi}{4}-\chi\right). \tag{S11}$$

This result indicates that the intensity of output light is solely dependent on the ellipticity parameter χ (the same cosine square relation of the conventional Malus' law), while the other parameter ψ remains an independent degree of freedom.

To derive the polarization projection relationship of light passing through an arbitrary PS polarizer [9] with allowed polarization state $|\alpha_0\ (2\psi_0,\ 2\chi_0)>$, and stopped state $|\alpha_0^{\perp}(2\psi_0+\pi,\ -2\chi_0)>$, we can first construct a local PS system whose north pole and south pole are $|\alpha_0>$ and $|\alpha_0^{\perp}>$, respectively, which play the same roles of the RCP ($|\alpha_{0l}\,(2\psi_{0l}=0,\ 2\chi_{0l}=\pi/2)>$) and LCP ($|\alpha_{0l}^{\perp}(2\psi_{0l}=0,\ 2\chi_{0l}=-\pi/2)>$) state in a global system, in terms of local PS parameters. Therefore, the Jones vector of an arbitrary polarization state $|\alpha_l(2\psi_l,\ 2\chi_l)>$ on the polarization base ($|\alpha_0>$, $|\alpha_0^{\perp}>$) is equivalent to the expression (Eq. S9) on the circular base ($|\alpha_{0l}>$, $|\alpha_{0l}^{\perp}>$) in terms of local PS parameters (ψ_l, χ_l), yielding

$$|\alpha_l(\psi_l,\chi_l)\rangle=\begin{bmatrix}<\alpha_{0l}|\alpha_l>\\<\alpha_{0l}^{\perp}|\alpha_l>\end{bmatrix}=\begin{bmatrix}\alpha_{RCP}\\\alpha_{LCP}\end{bmatrix}=A_0\exp(i\Phi)\begin{bmatrix}\cos(\pi/4-\chi_l)e^{i\psi_l}\\\sin(\pi/4-\chi_l)e^{-i\psi_l}\end{bmatrix}. \quad \text{(S12)}$$

Therefore, the projection of state $|\alpha_l\rangle$ on $|\alpha_{0l}\rangle$ yields the output intensity

$$I_{out}=|<\alpha_{0l}|\alpha_l>|^2=|\alpha_{RCP}|^2=A_0^2\cos^2(\pi/4-\chi_l). \quad \text{(S13)}$$

Note that, Eq. (S13) is the same as Eq. (1) in the main text. They are the derived GML expressed in terms of local PS parameters, which has a similar form as the conventional Malus' law.

To express the GML in the global PS system, we need to find the transformation relationship between the global PS coordinates (2ψ, 2χ) and the local PS coordinates ($2\psi_l$, $2\chi_l$) defined with respect to the polarization basis determined by the polarizer. The north pole of the local PS system $|\alpha_0\rangle$ has global PS coordinates ($2\psi_0$, $2\chi_0$) and local PS coordinates ($2\psi_{0l}$, $2\chi_{0l}$)=(0, π/2), respectively. To express local PS parameters by global PS parameters, we can rotate the global PS to coincide with the local PS. This operation is equivalent to rotating the global north pole point (0, π/2) to the local north pole point ($2\psi_0$, $2\chi_0$). The rotating angle can be determined by the PS coordinate difference between the global and local north pole, ($2\Delta\psi$, $2\Delta\chi$)=($2\psi_0$, $2\chi_0$-π/2). The rotation operation can be performed in two steps. First, rotating the PS by $\theta_y=2\Delta\chi$ angle with respect to the y (S_2)-axis, with the affine transformation matrix

$$\mathbf{R}_{2\Delta\chi}^{y}=\begin{pmatrix}\cos(2\Delta\chi) & 0 & -\sin(2\Delta\chi)\\0 & 1 & 0\\\sin(2\Delta\chi) & 0 & \cos(2\Delta\chi)\end{pmatrix}=\begin{pmatrix}\cos(2\chi_0-\frac{\pi}{2}) & 0 & -\sin(2\chi_0-\frac{\pi}{2})\\0 & 1 & 0\\\sin(2\chi_0-\frac{\pi}{2}) & 0 & \cos(2\chi_0-\frac{\pi}{2})\end{pmatrix}, \quad \text{(S14)}$$

and then, rotating by angle $\theta_z=2\psi_0$ with respect to z (S_3)-axis, with affine transformation matrix

$$\mathbf{R}_{2\Delta\psi}^{z}=\begin{pmatrix}\cos 2\Delta\psi & -\sin 2\Delta\psi & 0\\\sin 2\Delta\psi & \cos 2\Delta\psi & 0\\0 & 0 & 1\end{pmatrix}=\begin{pmatrix}\cos 2\psi_0 & -\sin 2\psi_0 & 0\\\sin 2\psi_0 & \cos 2\psi_0 & 0\\0 & 0 & 1\end{pmatrix}. \quad \text{(S15)}$$

The overall rotation operation can be written as the following affine transformation matrix

$$\mathbf{R}_{tot}=\mathbf{R}_{2\Delta\psi}^{z}\mathbf{R}_{2\Delta\chi}^{y}=\begin{pmatrix}\sin 2\chi_0\cos 2\psi_0 & -\sin 2\psi_0 & \cos 2\chi_0\cos 2\psi_0\\\sin 2\psi_0\sin 2\chi_0 & \cos 2\psi_0 & \cos 2\chi_0\sin 2\psi_0\\-\cos 2\chi_0 & 0 & \sin 2\chi_0\end{pmatrix}. \quad \text{(S16)}$$

As the above affine transformations matrices are defined in the Cartesian coordinate system, the local and global PS parameters should be transferred to Stokes parameters before operating the affine transformations. According to Eq. (S3), we can express global and local Stokes parameters $\mathbf{S}^g=(S_1^g,S_2^g,S_3^g)^T$, $\mathbf{S}^l=(S_1^l,S_2^l,S_3^l)^T$ in terms of PS parameters as

$$\begin{pmatrix}S_1^g\\S_2^g\\S_3^g\end{pmatrix}=\begin{pmatrix}\cos 2\chi\cos 2\psi\\\cos 2\chi\sin 2\psi\\\sin 2\chi\end{pmatrix},\quad\begin{pmatrix}S_1^l\\S_2^l\\S_3^l\end{pmatrix}=\begin{pmatrix}\cos 2\chi_l\cos 2\psi_l\\\cos 2\chi_l\sin 2\psi_l\\\sin 2\chi_l\end{pmatrix} \quad \text{(S17)}$$

The global and local Stokes parameters are connected by the following affine transformation,

$$\mathbf{S}^g=\mathbf{R}_{tot}\mathbf{S}^l, \quad \text{(S18)}$$

Substituting Eq. (S16) and Eq. (S17), in to Eq. (S18), we obtain,

$$\begin{aligned}\cos 2\chi \cos 2\psi &= \cos 2\chi_l \cos 2\psi_l \sin 2\chi_0 \cos 2\psi_0 \\ &-\cos 2\chi_l \sin 2\psi_l \sin 2\psi_0 + \sin 2\chi_l \cos 2\chi_0 \cos 2\psi_0\end{aligned}, \quad \text{(S19a)}$$

$$\begin{aligned}\cos 2\chi \sin 2\psi &= \cos 2\chi_l \cos 2\psi_l \sin 2\psi_0 \sin 2\chi_0 \\ &+\cos 2\chi_l \sin 2\psi_l \cos 2\psi_0 + \sin 2\chi_l \cos 2\chi_0 \sin 2\psi_0\end{aligned}, \quad \text{(S19b)}$$

$$\sin 2\chi = -\cos 2\chi_l \cos 2\psi_l \cos 2\chi_0 + \sin 2\chi_l \sin 2\chi_0 \,. \quad \text{(S19c)}$$

Then we can express the global PS parameters (ψ, χ) explicitly in terms of local PS parameters (ψ_l, χ_l), as well as the PS parameters of the polarizer (ψ_0, χ_0):

$$\psi = \frac{1}{2}\arctan\left[\frac{\cos 2\chi_l \cos 2\psi_l \sin 2\chi_0 \sin 2\psi_0 + \cos 2\chi_l \sin 2\psi_l \cos 2\psi_0 + \sin 2\chi_l \cos 2\chi_0 \sin 2\psi_0}{\cos 2\chi_l \cos 2\psi_l \sin 2\chi_0 \cos 2\psi_0 - \cos 2\chi_l \sin 2\psi_l \sin 2\psi_0 + \sin 2\chi_l \cos 2\chi_0 \cos 2\psi_0}\right], \quad \text{(S20a)}$$

$$\chi = \frac{1}{2}\arcsin\left[-\cos 2\chi_l \cos 2\psi_l \cos 2\chi_0 + \sin 2\chi_l \sin 2\chi_0\right]. \quad \text{(S20b)}$$

On the contrary, we can also express the local PS parameters (ψ_l, χ_l) in terms of local PS parameters (ψ, χ), as well as the PS parameters of the polarizer (ψ_0, χ_0), by reversing the affine transformation operation,

$$\mathbf{S}^l = \mathbf{R}_{tot}^{-1}\mathbf{S}^g \,, \quad \text{(S21)}$$

where

$$\mathbf{R}_{tot}^{-1} = \left(\mathbf{R}_{2\Delta\chi}^{y}\right)^{-1}\left(\mathbf{R}_{2\Delta\psi}^{z}\right)^{-1} = \begin{pmatrix} \sin 2\chi_0 \cos 2\psi_0 & \sin 2\chi_0 \sin 2\psi_0 & -\cos 2\chi_0 \\ -\sin 2\psi_0 & \cos 2\psi_0 & 0 \\ \cos 2\chi_0 \cos 2\psi_0 & \cos 2\chi_0 \sin 2\psi_0 & \sin 2\chi_0 \end{pmatrix}. \quad \text{(S22)}$$

Substituting Eq. (S17) and Eq. (S22) into Eq. (S21), we get,

$$\begin{aligned}&\cos 2\chi_l \cos 2\psi_l = \\ &\quad \sin 2\chi_0 \cos 2\psi_0 \cos 2\chi \cos 2\psi + \sin 2\chi_0 \sin 2\psi_0 \cos 2\chi \sin 2\psi - \cos 2\chi_0 \sin 2\chi\end{aligned}, \quad \text{(S23a)}$$

$$\cos 2\chi_l \sin 2\psi_l = -\sin 2\psi_0 \cos 2\chi \cos 2\psi + \cos 2\psi_0 \cos 2\chi \sin 2\psi \,, \quad \text{(S23b)}$$

$$\sin 2\chi_l = \cos 2\chi_0 \cos 2\psi_0 \cos 2\chi \cos 2\psi + \cos 2\chi_0 \sin 2\psi_0 \cos 2\chi \sin 2\psi + \sin 2\chi_0 \sin 2\chi \,. \quad \text{(S23c)}$$

Therefore, we obtain

$$\psi_l = \frac{1}{2}\arctan\left[\frac{\cos 2\chi \sin 2(\psi - \psi_0)}{\sin 2\chi_0 \cos 2\chi \cos 2(\psi - \psi_0) - \cos 2\chi_0 \sin 2\chi}\right], \quad \text{(S24a)}$$

$$\chi_l = \frac{1}{2}\arcsin\left[\cos 2\chi_0 \cos 2\chi \cos 2(\psi - \psi_0) + \sin 2\chi_0 \sin 2\chi\right]. \quad \text{(S24b)}$$

Substituting Eq. (S24b), into Eq. (S13), we obtain,

$$I_{out} = \frac{A_0^2}{2}[\cos 2\chi_0 \cos 2\chi \cos(2\psi - 2\psi_0) + \sin 2\chi_0 \sin 2\chi + 1]. \quad \text{(S25)}$$

Finally, we arrive at the closed from expression of GML that connects the output intensity of a

polarization state $|\alpha(2\psi, 2\chi)\rangle$ projected on another polarization state $|\alpha_0(\psi_0, \chi_0)\rangle$, both of which would be placed at arbitrary positions on the Poincare sphere surface. Note that for χ_0=0 and χ=0 Eq. (S25) collapses to the conventional Malus' law $I_{out} = A_0^2 \cos^2(\psi - \psi_0)$. Therefore, the conventional Malus' law is a special case of GML, which only describes the projection relationship of linear polarization states with vanishing helicities. In other words, the conventional Malus' law can be seen as a subset of the GML when the polarization trajectory is restricted in the equator of the PS, where only linear polarization states and linear polarizers are considered.

1.2. Further extension of generalized Malus' law to the solid surface Poincaré sphere

To discuss the most general case of the polarization projection phenomenon, both the incident light and the polarizer could be either fully or partially polarized, meaning that both of them could be located on arbitrary position of the *Solid* Poincare sphere characterized by a certain degree of polarization (DoP) [14]. Without loss of generality, we assume the polarization parameters (azimuth, ellipticity, DoP) of the partially polarized incident light and the partial polarizer are (ψ, χ, p), (ψ_0, χ_0, p_0), respectively. The partial polarizer could also be characterized by the Jones matrix in its local basis as,

$$J_p = \begin{pmatrix} 1 & 0 \\ 0 & c_0 \end{pmatrix}, \tag{S26}$$

Which represent that, its allowed state $|\alpha_0(\psi_0, \chi_0)\rangle$ has a transmission coefficient of unity, while the orthogonal state $|\alpha^{\perp}{}_0\rangle$ also have a transmission coefficient c_0, which connects the DoP of the polarizer by [14],

$$p_0 = \frac{1-|c_0|^2}{1+|c_0|^2} \text{ or } |c_0|^2 = \frac{1-p_0}{1+p_0}, \tag{S27}$$

As the incident partially polarized light (ψ, χ, p) can be considered as the combination of p proportion of fully polarized light and (1-p) proportion of unpolarized light, the polarization projection of (ψ, χ, p) on (ψ_0, χ_0, p_0) can be divided into two parts: 1. The projection from unpolarized light to the partial polarizer; 2. The projection from the full polarized light to the polarizer.

1. When a beam of unpolarized light with unitary power passes through the partial polarizer, the output light become a partial polarized light composed of a portion of unpolarized light of power,

$$P_{1u} = |c_0|^2, \tag{S28}$$

and a portion of fully polarized light of power,

$$P_{1p} = (1 - |c_0|^2)/2, \tag{S29}$$

Therefore, the total output power of the projected partial polarized light is,

$$P_{1total} = P_{1u} + P_{1p} = (1 + |c_0|^2)/2, \tag{S30}$$

Substituting Eq. (2) in to Eq. (5), we obtain,

$$P_{1total} = 1/(1 + p_0), \tag{S31}$$

Therefore, (1-p) portion of unpolarized light in the incident light will produce output intensity of,

$$P_1 = (1 - p)/(1 + p_0), \tag{S32}$$

2. When the full polarized light $|\alpha(\psi_l, \chi_l)\rangle$ with unity power, is incident upon the same partially polarizing element, the resulting light intensity, denoted as I_p, can be obtained by the Jones matrix calculation in the local circular polarization basis.

$$\alpha_p = J_p|\alpha_l(\psi_l,\chi_l)> = \begin{pmatrix}1 & 0\\ 0 & c_0\end{pmatrix}\exp(i\Phi)\begin{bmatrix}cos\left(\frac{\pi}{4}-\chi_l\right)e^{i\psi_l}\\ sin\left(\frac{\pi}{4}-\chi_l\right)e^{-i\psi_l}\end{bmatrix} = \exp(i\Phi)\begin{pmatrix}cos(\pi/4-\chi_l)\,e^{i\psi_l}\\ c_0\,sin(\pi/4-\chi_l)\,e^{-i\psi_l}\end{pmatrix}, \quad \text{(S33)}$$

Therefore, the transmitted intensity can be written as

$$p_{2total} = cos^2(\pi/4-\chi_l) + |c_0|^2\, sin^2(\pi/4-\chi_l), \quad \text{(S34)}$$

Therefore, p portion of fully polarized light will produce output light of power,

$$p_2 = p * p_{2total} = p\left[cos^2\left(\frac{\pi}{4}-\chi_l\right) + \frac{1-p_0}{1+p_0}sin^2\left(\frac{\pi}{4}-\chi_l\right)\right], \quad \text{(S35)}$$

Note that, Eq. (S35) represents the projection from a polarized light to a partial polarizer, which was consistent with some previous work on the projection involving imperfect polarizers[15], which is only a small subset of our present GML at the most general case.

Therefore, the total output power from the partial polarized light to the partial polarizer in the local Poincare sphere system yields,

$$I_{out} = A_0^2(p_1+p_2) = \frac{1-p}{1+p_0} + p\left[cos^2\left(\frac{\pi}{4}-\chi_l\right) + \frac{1-p_0}{1+p_0}sin^2\left(\frac{\pi}{4}-\chi_l\right)\right] = A_0^2\frac{1-pp_0}{1+p_0} + A_0^2\frac{2pp_0}{1+p_0}cos^2\left(\frac{\pi}{4}-\chi_l\right), \quad \text{(S36)}$$

where, A_0 is the amplitude of the incident light. This is the local form of the *solid* PS GML. To transfer the local form to the global form, affine transformation between the local and global coordinates, we finally obtain the global form of the *solid* PS GML as,

$$I_{out} = A_0^2\frac{1-pp_0}{1+p_0} + A_0^2\frac{pp_0}{1+p_0}[cos2\chi_0 cos2\chi cos(2\psi-2\psi_0) + sin2\chi_0 sin2\chi + 1], \quad \text{(S37)}$$

When both p and p_0 equal 1, it corresponds to the scenario where fully polarized light is incident upon a perfectly polarizing element, agree with Eq. (S25).

2. Detailed design procedure for the Poincare sphere trajectory/grid encoding and decoding.

The detailed design procedure to modulate a polarization image superposed with an arbitrary analytic PS trajectory is shown in Supplementary Fig. 9. Each pixel of the image is modulated by the local ellipticity parameter χ_l according to the local form of GML as

$$\chi_l(x,y) = \pi/4 - \arccos\sqrt{I(x,y)}\,. \quad \text{(S38)}$$

The local azimuth parameter ψ_l is assigned by an analytic function with respect to χ_l, manifesting a modulation trajectory on the PS. The modulated single image together with the analytic function form of the modulation trajectory provide complete coordinate information on the PS, namely, the local PS parameters (ψ_l, χ_l). Additionally with the information of polarization basis parameters (ψ_0, χ_0), the global PS parameters (ψ, χ) can be determined by Eq. (20).

After we obtain the global PS parameters (ψ, χ), we can map the polarization parameters into the meta-atom response parameters ($\Delta\varphi$, θ) according to Eq. (S50), and then determine the meta-atom structure parameters (D_x, D_y, θ) by searching the structure parameter library color-map provided by Supplementary Fig. 7 and 8.

In the decoding process, a circularly polarized incident light is employed to illuminate the encoded metasurface, a spatially varying polarization profile will be generated on the transmitted beam. Applying the global form of GML, the polarization profile will be transformed into a certain intensity pattern as the output image. By varying the polarization state of the decoding analyzer

along a certain path on the PS, different grayscale-adjusted version of the encoded image will be analyzed.

The detailed design procedure for a double information channel encryption based on two local PS systems is shown in Supplementary Fig. 11. First, we choose two arbitrary sets of local PS systems with polarization basis parameters (ψ_0, χ_0) and (ψ_0', χ_0') for local PS I and PS II, respectively, which means that the polarization sates for the local north/south poles of PS I are $|\alpha_0(2\psi_0, 2\chi_0)\rangle$ and $|\alpha_0^{\perp}(2\psi_0+\pi, -2\chi_0)\rangle$, and that of the PS II are $|\alpha_0'(2\psi_0', 2\chi_0')\rangle$ and $|\alpha_0'^{\perp}(2\psi_0'+\pi, -2\chi_0')\rangle$, respectively. Then, we modulate two independent sets of spatially varying polarization information represented by two grayscale images 'A' and 'B' into local PS I and II, respectively. According to the local form of GML, two sets of local ellipticity parameters $\chi_l(x, y)$ and $\chi_l'(x, y)$ can be directly determined from the intensity value of the two image,

$$\chi_l(x,y) = \pi/4 - \arccos\sqrt{I_A(x,y)}, \tag{S39a}$$

$$\chi_l'(x,y) = \pi/4 - \arccos\sqrt{I_B(x,y)}, \tag{S39b}$$

Based on the above two sets of local ellipticity parameters of two local PS systems with given basis PS parameters, we are supposed to determine the global PS parameters (ψ, χ). According to the transformation relation between local PS parameters, global PS parameters and basis PS parameters provided by Eq. (S23c), we obtain,

$$\sin 2\chi_l = \cos 2\chi_0 \cos 2\psi_0 \cos 2\chi \cos 2\psi + \cos 2\chi_0 \sin 2\psi_0 \cos 2\chi \sin 2\psi + \sin 2\chi_0 \sin 2\chi, \tag{S40a}$$

$$\sin 2\chi_l' = \cos 2\chi_0' \cos 2\psi_0' \cos 2\chi \cos 2\psi + \cos 2\chi_0' \sin 2\psi_0' \cos 2\chi \sin 2\psi + \sin 2\chi_0' \sin 2\chi, \tag{S40b}$$

Now, we have two independent equations for two unknown parameters (ψ, χ). Therefore, the global PS parameters are uniquely determined. According to the GML, the decode images of 'A' and 'B' appears at the local north poles of local PS I and PS II respectively, namely, $|\alpha_0(2\psi_0, 2\chi_0)\rangle$ and $|\alpha_0'(2\psi_0', 2\chi_0')\rangle$, which could be designed at two arbitrary locations on the PS in principle. It indicates that, multiplexed two independent information channels are not necessarily orthogonal, but could be arbitrary assigned. Based on this principle, one could not only encrypt the information content itself, but also encrypt the locations of the information channels arbitrarily on the PS, largely enhancing the encryption security level.

The general solutions for (ψ, χ) based on Eqs. (S41) are complex. At some special cases, the explicit solution can be significantly simplified. Consider a particular situation when the local north/south poles of one local PS are set at the equator of the other local PS, so that the two sets of local latitudes are perpendicular to each other. To simplify the solving process, we first transform all global PS parameters to local parameters on PS I. After the calculation in terms of local parameters on PS I, we transform them back into global PS parameters.

Under the local PS I, the corresponding local parameters for the polarization basis parameter (ψ_0, χ_0) of PS I itself are the parameters for RCP state: (ψ_{0l}, χ_{0l})=(0, 45°); and the corresponding local parameters (ψ_{0l}', χ_{0l}') for the polarization basis parameter (ψ_0', χ_0') of PS II can be obtained according to the global to local transform relation (Eq. 24) as follows,

$$\psi_{0l}' = \frac{1}{2}\arctan\left[\frac{\cos 2\chi_0' \sin 2(\psi_0' - \psi_0)}{\sin 2\chi_0 \cos 2\chi_0' \cos 2(\psi_0' - \psi_0) - \cos 2\chi_0 \sin 2\chi_0'}\right], \tag{S41a}$$

$$\chi_{0l}'=\frac{1}{2}\arcsin\left[\cos 2\chi_0\cos 2\chi_0'\cos 2(\psi_0'-\psi_0)+\sin 2\chi_0\sin 2\chi_0'\right]. \tag{S41b}$$

Assuming that the polarization basis poles of local PS II are lying on the equator of the local PS I, it requires χ_{0l}'=0. Therefore, under the local PS I, we do the following variable substitution: (ψ_0, χ_0) -> (ψ_{0l}, χ_{0l})=(0, 45°) , (ψ_0', χ_0') -> (ψ_{0l}', χ_{0l}')=(ψ_{0l}', 0), (ψ, χ) -> (ψ_{PS1}, χ_{PS1}), where the subscript PS1 denote that the solved parameters are local parameters on PS I, Eqs. S40 can be simplified as follows,

$$\sin 2\chi_l=\sin 2\chi_{PS1}, \tag{S42a}$$

$$\sin 2\chi_l'=\left(\cos 2\psi_{0l}'\cos 2\psi_{PS1}+\sin 2\psi_{0l}'\sin 2\psi_{PS1}\right)\cos 2\chi_{PS1}, \tag{S42b}$$

Therefore,

$$\chi_{PS1}=\chi_l, \tag{S43a}$$

$$\psi_{PS1}=\frac{1}{2}\arccos\frac{\sin 2\chi_l'}{\cos 2\chi_l}+\psi_{0l}', \tag{S43b}$$

Then we can transfer the local parameters on PS I (ψ_{PS1}, χ_{PS1}) to global parameters according to Eqs. (S20) as

$$\psi=\frac{1}{2}\arctan\left[\frac{\left(\begin{array}{c}\cos 2\chi_l\cos(\arccos\frac{\sin 2\chi_l'}{\cos 2\chi_l}+2\psi_{0l}')\sin 2\chi_0\sin 2\psi_0+\\ \cos 2\chi_l\sin(\arccos\frac{\sin 2\chi_l'}{\cos 2\chi_l}+2\psi_{0l}')\cos 2\psi_0+\sin 2\chi_l\cos 2\chi_0\sin 2\psi_0\end{array}\right)}{\left(\begin{array}{c}\cos 2\chi_l\cos(\arccos\frac{\sin 2\chi_l'}{\cos 2\chi_l}+2\psi_{0l}')\sin 2\chi_0\cos 2\psi_0-\\ \cos 2\chi_l\sin(\arccos\frac{\sin 2\chi_l'}{\cos 2\chi_l}+2\psi_{0l}')\sin 2\psi_0+\sin 2\chi_l\cos 2\chi_0\cos 2\psi_0\end{array}\right)}\right], \tag{S44a}$$

$$\chi=\frac{1}{2}\arcsin\left[-\cos 2\chi_l\cos(\arccos\frac{\sin 2\chi_l'}{\cos 2\chi_l}+2\psi_{0l}')\cos 2\chi_0+\sin 2\chi_l\sin 2\chi_0\right], \tag{S44b}$$

particularly when one local PS (PS I) is set under the circular polarization basis (which is coincident with the global PS system), and the other PS (PS II) is set under the linear polarization basis with north pole (ψ_0'=0, χ_0'=0), the solutions for (ψ, χ) can be further simplified as follows,

$$\chi=\chi_l, \tag{S45a}$$

$$\psi=\frac{1}{2}\arccos\frac{\sin 2\chi_l'}{\cos 2\chi_l}, \tag{S45b}$$

After the determination of global PS parameters, the following encoding steps for meta-atom parameter mapping and the whole decoding process are exactly the same as that for the analytical PS trajectory modulation.

3. Explicit modulation relations between metasurface parameters and the generated polarizations covering the full Poincare sphere

Employing metasurface to manipulate optical field point by point, each meta-atom could be typically written as the following Jones matrix [16-18]

$$J = AR(\theta)\begin{pmatrix} e^{i\varphi_x} & 0 \\ 0 & e^{i\varphi_y} \end{pmatrix} R(-\theta), \tag{S46}$$

where A is the modulated amplitude; φ_x, φ_y, θ are phase retardations along two eigen-polarization directions and the orientation angle of the meta-atom; $R(\theta)=\begin{pmatrix} \cos\theta & -\sin\theta \\ \sin\theta & \cos\theta \end{pmatrix}$ is the rotation matrix.

By setting $\Delta\varphi=(\varphi_x-\varphi_y)/2$, $\varphi=(\varphi_x+\varphi_y)/2$, we can rewrite the Jones matrix as

$$\boldsymbol{J} = Ae^{i\varphi}\begin{pmatrix} e^{i\Delta\varphi} cos^2\theta + e^{-i\Delta\varphi} sin^2\theta & (e^{i\Delta\varphi} - e^{-i\Delta\varphi}) \cos\theta \sin\theta \\ (e^{i\Delta\varphi} - e^{-i\Delta\varphi}) \cos\theta \sin\theta & e^{i\Delta\varphi} sin^2\theta + e^{-i\Delta\varphi} cos^2\theta \end{pmatrix}, \tag{S47}$$

where $Ae^{i\varphi}$ is the overall complex amplitude factor, which does not affect the polarization states, therefore only the parameters Δφ and θ relate the modulated polarization states of light.

To obtain the direct mapping relation between the metasurface parameters ($\Delta\varphi$, θ) and the polarization PS parameters (ψ, χ), It is convenient to transfer the Jones matrix from the linear base $\mathbf{J}$ to its circular base form $\mathbf{J}^C$,

$$\boldsymbol{J}^C = \boldsymbol{\Lambda}^{-1}\boldsymbol{J}\boldsymbol{\Lambda} = Ae^{i\varphi}\begin{pmatrix} cos\Delta\varphi & sin\Delta\varphi e^{i(2\theta+\frac{\pi}{2})} \\ sin\Delta\varphi e^{-i(2\theta-\frac{\pi}{2})} & cos\Delta\varphi \end{pmatrix}. \tag{S48}$$

Under RCP incidence (linear base: $\boldsymbol{\alpha}_{RCP}=\frac{\sqrt{2}}{2}\begin{pmatrix} 1 \\ -i \end{pmatrix}$, circular base: $\boldsymbol{\alpha}^C_{RCP}=\begin{pmatrix} 1 \\ 0 \end{pmatrix}$), the output Jones vector on circular base are

$$\begin{aligned}\boldsymbol{\alpha}^C = \boldsymbol{J}^C\boldsymbol{\alpha}^C_{RCP} &= Ae^{i\varphi}\begin{pmatrix} cos\Delta\varphi & sin\Delta\varphi e^{i(2\theta+\frac{\pi}{2})} \\ sin\Delta\varphi e^{-i(2\theta-\frac{\pi}{2})} & cos\Delta\varphi \end{pmatrix}\begin{pmatrix} 1 \\ 0 \end{pmatrix} \\ &= Ae^{i\varphi}e^{-i(\theta-\frac{\pi}{4})}\begin{pmatrix} cos\Delta\varphi e^{i(\theta-\frac{\pi}{4})} \\ sin\Delta\varphi e^{-i(\theta-\frac{\pi}{4})} \end{pmatrix}.\end{aligned} \tag{S49}$$

comparing Eq. (S49) and Eq. (S9), we obtain

$$\Delta\varphi = \frac{\pi}{4} - \chi,\ \theta = \psi + \frac{\pi}{4}. \tag{S50}$$

For a given polarization state (ψ, χ), we can readily generate it by a meta-atom with response parameters ($\Delta\varphi$, θ) under circular polarization illumination, providing a versatile full polarization encoding approach beyond pure linear polarizations. In practice, parameter Δφ can be determined by the length and width of the meta-atom (D_x, D_y), and parameter θ is determined by the orientation angle of the meta-atom, as the standard design procedure of all-dielectric metasurfaces [19-25].

In our practical experimental implementation, we employed all-dielectric metasurfaces composed of silicon anisotropic nano-pillars as meta-atoms standing on silica substrate. The meta-atoms are fully covered by a SU8 protective layer, as shown in Supplementary Fig. 7a. We target at the near-infrared wavelength λ_0=940nm, which is widely applicable in many application fields such

as light detection and ranging, facial recognition with 3D structure light, and automatic drive. The period p and thickness h of the meta-atoms are fixed as 500 nm, and 670 nm. The length D_x and width D_y width of the meta-atom are varied to modulate the anisotropic phase φ_x and φ_y. The corresponding relation between D_x, D_y and φ_x, φ_y are showing in Supplementary Fig. 7c and 7d, which can serve as a complete library for searching meta-atom sizes. The transmission efficiencies with the same meta-atom parameter library are shown in Supplementary Fig. 7e and 7f. In most area, the transmission generally has high values close to 1, except for some discrete sharp dips due to resonances [26]. In the practical determination of the meta-atom length and width, the resonance dips can be avoided by choosing other parameters as there are multiple D_x, D_y corresponding to a particular set of φ_x, φ_y .

In practical encoding an image by the metasurface, as only $\Delta\varphi$ (not necessarily be aware of the value of both φ_x, φ_y) is need for modulation, one need only search D_x and D_y directly on the colormap of Δφ as shown in Figs. S8a, d and g. For the linear polarization basis metasurfaces, we only need to find one point with Δφ=π/4 (as a λ/4 waveplate) [27] with D_x=300nm, D_y=170nm, as shown in Supplementary Fig. 8d. For the circular polarization basis, we quantize the image to multiple discrete levels, with corresponding D_x, and D_y denoted by the triangles in Figs. S8a-c and g-i. At the searched points, the transmission resonant dips are carefully avoided, as shown in Supplementary Fig. 8b, e, h and c, f, i, to promise the high transmission efficiencies of the metasurface.

In the modulation of polarization state, the intensity of the incident laser light beam can be deemed as uniform, and therefore the Stokes parameters are directly modulated by the phase retardation (Δφ=φx-φy) and orientation angle (θ) of the anisotropic meta-atom, without normalizing the values of the Stokes parameters by the total intensity. For the output images, the encoded polarization information is transformed to intensity information after the polarization analyzer. Therefore, the final decoded output image is directly captured by the CCD after the analyzer, hence there is no need for normalizing the Stokes parameters.

4. Construct of the overall Poincare sphere analyzer with GML by cascading a 1/4 waveplate and a linear polarizer.

At the linear base, the Jones matrix of a 1/4 waveplate and a linear polarizer are, respectively

$$\begin{aligned} J_{\lambda/4} &= R(\theta_{\lambda/4})\begin{pmatrix}1 & 0\\ 0 & i\end{pmatrix}R(-\theta_{\lambda/4}) \\ &= \begin{pmatrix}\cos\theta_{\lambda/4} & -\sin\theta_{\lambda/4}\\ \sin\theta_{\lambda/4} & \cos\theta_{\lambda/4}\end{pmatrix}\begin{pmatrix}1 & 0\\ 0 & i\end{pmatrix}\begin{pmatrix}\cos\theta_{\lambda/4} & \sin\theta_{\lambda/4}\\ -\sin\theta_{\lambda/4} & \cos\theta_{\lambda/4}\end{pmatrix} \\ &= \begin{pmatrix}\cos^2\theta_{\lambda/4} + i\sin^2\theta_{\lambda/4} & \sin\theta_{\lambda/4}\cos\theta_{\lambda/4} - i\sin\theta_{\lambda/4}\cos\theta_{\lambda/4}\\ \sin\theta_{\lambda/4}\cos\theta_{\lambda/4} - i\sin\theta_{\lambda/4}\cos\theta_{\lambda/4} & \sin^2\theta_{\lambda/4} + i\cos^2\theta_{\lambda/4}\end{pmatrix} \end{aligned}, \quad \text{(S51)}$$

$$\begin{aligned} J_p &= R(\theta_p)\begin{pmatrix}1 & 0\\ 0 & 0\end{pmatrix}R(-\theta_p) \\ &= \begin{pmatrix}\cos\theta_p & -\sin\theta_p\\ \sin\theta_p & \cos\theta_p\end{pmatrix}\begin{pmatrix}1 & 0\\ 0 & 0\end{pmatrix}\begin{pmatrix}\cos\theta_p & \sin\theta_p\\ -\sin\theta_p & \cos\theta_p\end{pmatrix}. \\ &= \begin{pmatrix}\cos^2\theta_p & \cos\theta_p\sin\theta_p\\ \cos\theta_p\sin\theta_p & \sin^2\theta_p\end{pmatrix} \end{aligned} \quad \text{(S52)}$$

where, $\theta_{\lambda/4}$ and θ_p are the rotating angle of the 1/4 waveplate and the polarizer, respectively.

Transform them into the circular basis, yielding,

$$\mathbf{J}_{\lambda/4}^{C}=\mathbf{\Lambda}^{-1}\mathbf{J}_{\lambda/4}\mathbf{\Lambda}=\frac{\sqrt{2}}{2}\begin{pmatrix} \mathrm{e}^{i\frac{\pi}{4}} & e^{i(2\theta_{\lambda/4}-\frac{\pi}{4})} \\ e^{-i(2\theta_{\lambda/4}+\frac{\pi}{4})} & \mathrm{e}^{i\frac{\pi}{4}} \end{pmatrix}, \tag{S53}$$

$$\mathbf{J}_{p}^{C}=\mathbf{\Lambda}^{-1}\mathbf{J}_{p}\mathbf{\Lambda}=\frac{1}{2}\begin{pmatrix} 1 & e^{i2\theta_p} \\ e^{-i2\theta_p} & 1 \end{pmatrix}, \tag{S54}$$

Cascading of a 1/4 waveplate with rotating angle $\theta_{\lambda/4}$ and a linear polarizer with rotating angle θ_p forms a Poincare sphere analyzer that can filter out an arbitrary polarization state $|\alpha_0(2\psi_0, 2\chi_0)>$, which completely stop its orthogonal state$|\alpha_0^{\perp}(2\psi_0+\pi, -2\chi_0)>$. To determine the required ($\theta_{\lambda/4}$, θ_p) combination for a given analyzed polarization parameters $|\alpha_0(2\psi_0, 2\chi_0)>$, we can reverse the order of the 1/4 waveplate and the polarizer. That is, let the incident first go through the polarizer and then the 1/4 waveplate and set the final output polarization as the preset polarization $|\alpha_0(2\psi_0, 2\chi_0)>$. After the incident light go through the linear polarizer, no matter what polarization state the incident light is, the output light should always be a linear polarization with the same orientation angle θ_p of the polarizer. Its normalized Jones vector can be written at the linear base as,

$$\boldsymbol{\alpha}_p=\begin{pmatrix} \cos\theta_p \\ \sin\theta_p \end{pmatrix}, \tag{S55}$$

and it can be written under the circular basis as

$$\boldsymbol{\alpha}_p^{C}=\mathbf{\Lambda}^{-1}\boldsymbol{\alpha}_p=\frac{\sqrt{2}}{2}\begin{pmatrix} e^{i\theta_p} \\ e^{-i\theta_p} \end{pmatrix}. \tag{S56}$$

Then after it further goes through the 1/4 waveplate, the final output light should be,

$$\boldsymbol{\alpha}_{p\text{->}\lambda/4}^{C}=\mathbf{J}_{\lambda/4}^{C}\boldsymbol{\alpha}_p^{C}=\mathrm{e}^{i\frac{\pi}{4}}\begin{pmatrix} \cos\left(\theta_p-\theta_{\lambda/4}+\frac{\pi}{4}\right)e^{i\theta_{\lambda/4}} \\ \sin\left(\theta_p-\theta_{\lambda/4}+\frac{\pi}{4}\right)e^{-i\theta_{\lambda/4}} \end{pmatrix}. \tag{S57}$$

Ignoring the overall phase factor $\mathrm{e}^{i\frac{\pi}{4}}$, and comparing Eq.(S9), we can build the following equation

$$\begin{pmatrix} \cos\left(\theta_p-\theta_{\lambda/4}+\frac{\pi}{4}\right)e^{i\theta_{\lambda/4}} \\ \sin\left(\theta_p-\theta_{\lambda/4}+\frac{\pi}{4}\right)e^{-i\theta_{\lambda/4}} \end{pmatrix}=\begin{pmatrix} \cos\left(\frac{\pi}{4}-\chi_0\right)e^{i\psi_0} \\ \sin\left(\frac{\pi}{4}-\chi_0\right)e^{-i\psi_0} \end{pmatrix}, \tag{S58}$$

therefore

$$\psi_0=\theta_{\lambda/4},\ \chi_0=\theta_{\lambda/4}-\theta_p, \tag{S59}$$

or

$$\theta_{\lambda/4}=\psi_0,\ \theta_p=\psi_0-\chi_0. \tag{S60}$$

Substituting Eq. (S60) into Eq. (S53) and Eq. (S54), we can build the Jones matrix of the Poincare sphere polarizer for the allowed polarization state (ψ_0, χ_0) as,

$$\mathbf{J}_{PSP}^{C}\left(\psi_0,\chi_0\right)=\mathbf{J}_{p}^{C}\mathbf{J}_{\lambda/4}^{C}=\frac{\sqrt{2}}{2}\begin{pmatrix}\cos\left(\chi_0-\frac{\pi}{4}\right)e^{i\chi_0} & \cos\left(\chi_0+\frac{\pi}{4}\right)e^{i(2\psi_0+\chi_0)}\\ \cos\left(\chi_0-\frac{\pi}{4}\right)e^{-i(2\psi_0+\chi_0)} & \cos\left(\chi_0+\frac{\pi}{4}\right)e^{-i\chi_0}\end{pmatrix}, \tag{S61}$$

Under illumination with arbitrary incident polarization state (ψ, χ), the output Jones vector is

$$\boldsymbol{\alpha}_{out}^{C}=\mathbf{J}_{PSP}^{C}\left(\psi_0,\chi_0\right)\boldsymbol{\alpha}_{in}^{C}\left(\psi,\chi\right)=\begin{pmatrix}\alpha_{out}^{RCP}\\ \alpha_{out}^{LCP}\end{pmatrix}$$

$$=\frac{\sqrt{2}}{2}\begin{pmatrix}\cos\left(\chi_0-\frac{\pi}{4}\right)\cos\left(\frac{\pi}{4}-\chi\right)e^{i(\chi_0+\psi)}+\cos\left(\chi_0+\frac{\pi}{4}\right)\sin\left(\frac{\pi}{4}-\chi\right)e^{i(2\psi_0+\chi_0-\psi)}\\ \cos\left(\chi_0-\frac{\pi}{4}\right)\cos\left(\frac{\pi}{4}-\chi\right)e^{i(-2\psi_0-\chi_0+\psi)}+\cos\left(\chi_0+\frac{\pi}{4}\right)\sin\left(\frac{\pi}{4}-\chi\right)e^{i(-\chi_0-\psi)}\end{pmatrix}, \tag{S62}$$

In this way, we can also obtain the output intensity of the Poincare sphere polarizer as,

$$I_{out}=\left|\alpha_{out}^{RCP}\right|^2+\left|\alpha_{out}^{LCP}\right|^2=\frac{1}{2}[\cos 2\chi_0\cos 2\chi\cos(2\psi-2\psi_0)+\sin 2\chi_0\sin 2\chi+1], \tag{S63}$$

which is consistent with Eq. (S25), as the final expression of the GML.

Supplementary Figures

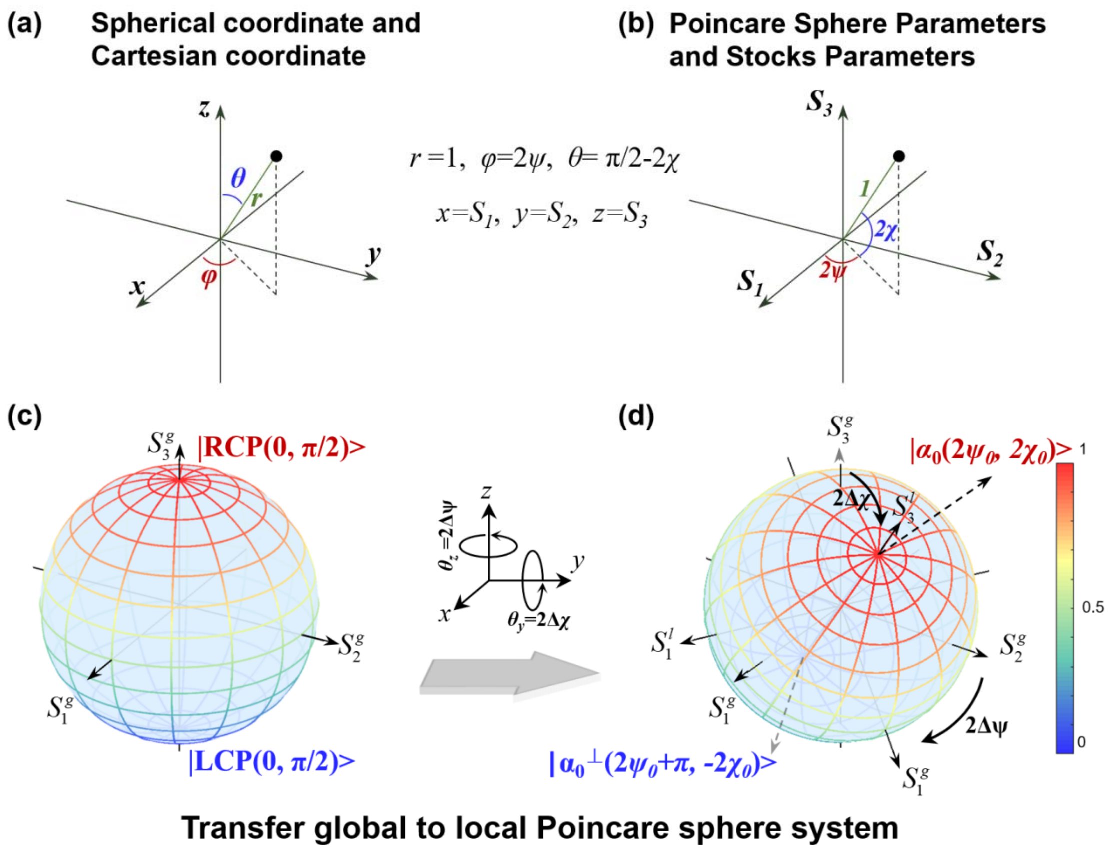

Supplementary Fig. 1. Affine transformation between the global PS coordinate and the local PS coordinate. (a) Global PS systems with global Stokes parameters (S_1^g, S_2^g, S_3^g), the polarization base vectors are |RCP> and |LCP>, which correspond to the north and south poles of the PS. The GML governed output intensities are constants along each latitude lines, while follow the same cosine square modulation relation along each longitudinal lines, as the line colors indicate. (b) Rotating the coordinate axes of the global PS system by θ_y=2$\Delta\chi$ with respect to y-axis, and by θ_z =2$\Delta\psi$ with respect to the z-axis, the global PS system will be transformed to a local PS system with polarization base vectors $|\alpha_0(2\psi_0, 2\chi_0)>$ and $|\alpha_0^{\perp}(2\psi_0+\pi, -2\chi_0)>$, where, $(2\Delta\psi, 2\Delta\chi)=(2\Delta\psi, 2\chi_0-\pi/2)$ are the coordinate differences between the circular polarization base vectors (|RCP>, |LCP>) and the local polarization base vectors ($|\alpha_0>$, $|\alpha_0^{\perp}>$). In the local PS system represented by local Stokes parameters (S_1^l, S_2^l, S_3^l), the polarization base vectors plays the same roles of the circular base vectors, namely, $|\alpha_{0l}>=|RCP_l>$, $|\alpha_{0l}^{\perp}>=|LCP_l>$. It means that the polarization base can be considered as a pair of local north/south poles, forming a local network of latitude and longitudinal lines that represent the iso-intensity and cosine-square modulation paths under an arbitrary polarization base ($|\alpha_0>$, $|\alpha_0^{\perp}>$).

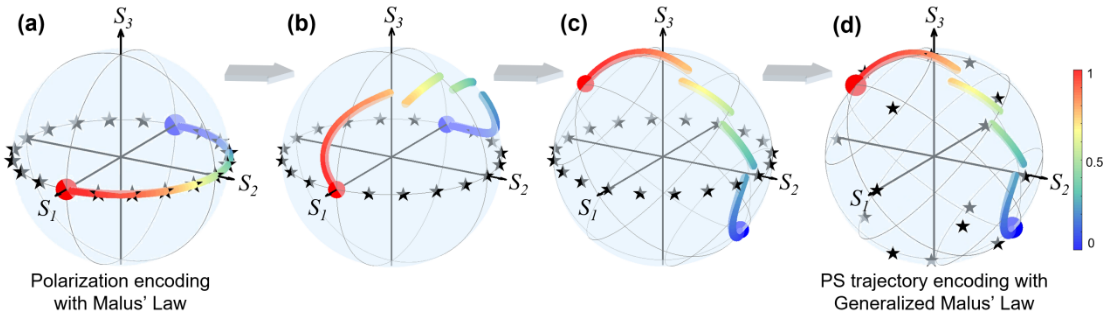

Supplementary Fig. 2. Evolution of polarization encoding with Malus' Law to Poincare sphere trajectory encoding with Generalized Malus' law. The red/blue dots are the local north/south poles of the local Poincare sphere system, representing the polarizer allowed and stopped states, respectively. The black stars represent the analyzer locations to decode the image. The colored curves represent the modulation trajectory, where the color denotes the modulated intensity on the corresponding location of the Poincare sphere. (a) For polarization encoding with Malus' law, both the encoding trajectory and decoding analyzer locations are restricted to the equator of the Poincare sphere. For Poincare sphere trajectory encoding with generalized Malus' law, (b) the modulation trajectory can be released from the equator of the Poincaré sphere to an arbitrary path connecting the polarization basis poles as denoted by the red and blue dots. (c) The polarization basis pole positions can also be released from the equator of the Poincaré sphere to any arbitrary positions on the Poincaré sphere. (d) The decoding analyzer positions could also be distributed any positions of the Poincare sphere rather than being restricted on the equator of the Poincaré sphere. (d) Shows the most general case of the Poincaré sphere trajectory encoding and decoding process with extended possibility of modulation trajectory, encoding polarization bases and decoding analyzer positions.

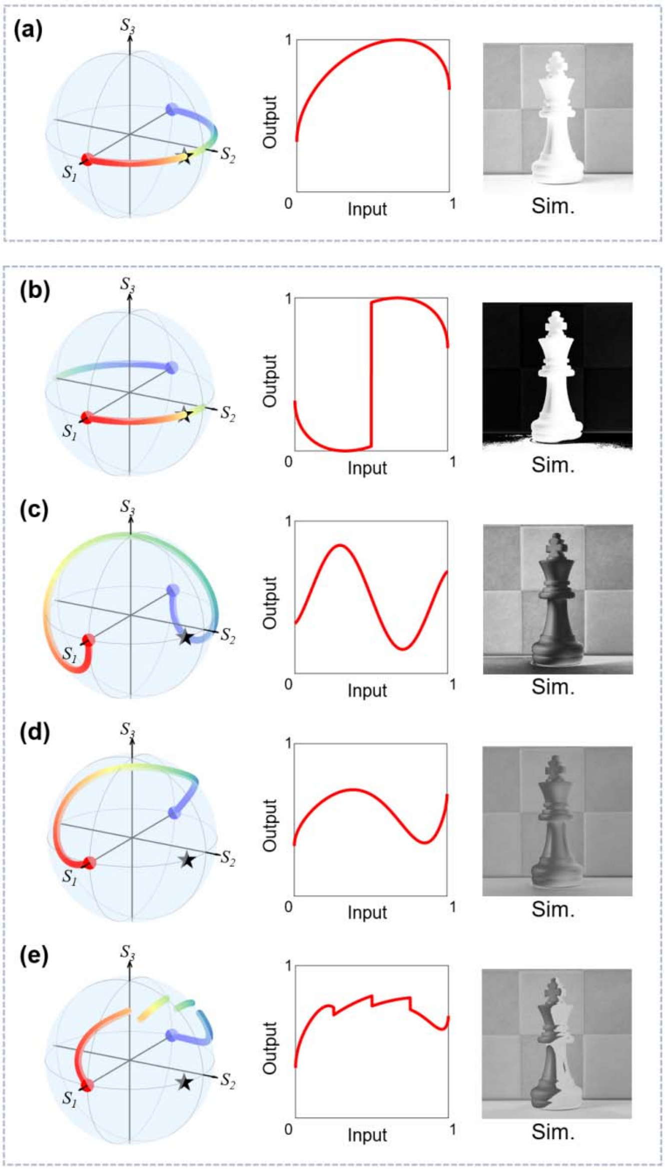

Supplementary Fig. 3. Comparison of the diversity of input-ouput relation and output images between (a) polarization encoding based on conventional Malus' law and (b-e) PS trajectory encoding based on generalized Malus law.

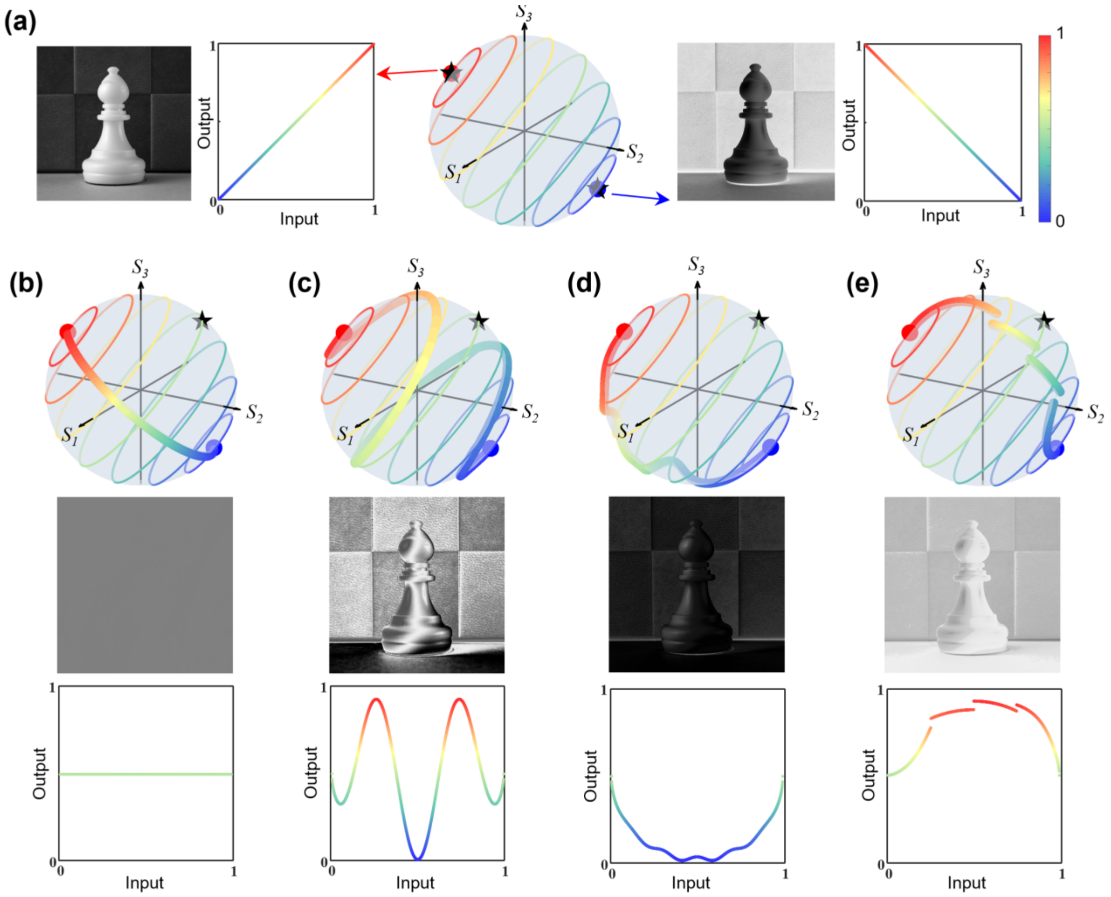

Supplementary Fig. 4. Image encoding/decoding with the same polarization basis poles and analyzing location, but different PS trajectories based on GML. (a) Encoding an image on a polarization basis poles ($|\alpha_0\rangle$, $|\alpha_0^{\perp}\rangle$) denoted by the red and blue dots on the PS maps each image intensity value on the latitude lines defined on the local PS system, making infinite number of encoding possibilities as the modulation point for each intensity sweeps an entire circle. No matter which point on the latitude line is chosen, the original (left) and grayscale-inversed (right) image are always decoded at the $|\alpha_0\rangle$ and $|\alpha_0^{\perp}\rangle$ state, respectively. (b-e) by choosing different PS modulation trajectories (b) $\psi_l = f(\chi_l) = \pi/4$, (c) $\psi_l = 4\cos^2(\chi_l - \pi/4)$ (d) $\psi_l = 10 sin(5\pi\cos^2(\chi_l - \pi/4))$ (e) $\psi_l = 5^{\cos 2(\chi_l - \pi/4)+1} - 7\pi/9$ ($-\pi/4 \le \chi_l < -\pi/12$); $\psi_l = 5\pi/12$ ($-\pi/12 \le \chi_l < 0$); $\psi_l = \pi/3$ ($0 \le \chi_l < \pi/12$); $\psi_l = 6^{\cos 2(\chi_l - \pi/4)+1} + 5\pi/12$ ($\pi/12 \le \chi_l \le \pi/4$), the decoded image information (middle panels) is different at a analyzer state (denoted by the black star) other than the polarization basis states $|\alpha_0\rangle$ and $|\alpha_0^{\perp}\rangle$. The corresponding histogram transfer function (lowest panels) can well be engineered by the PS trajectory.

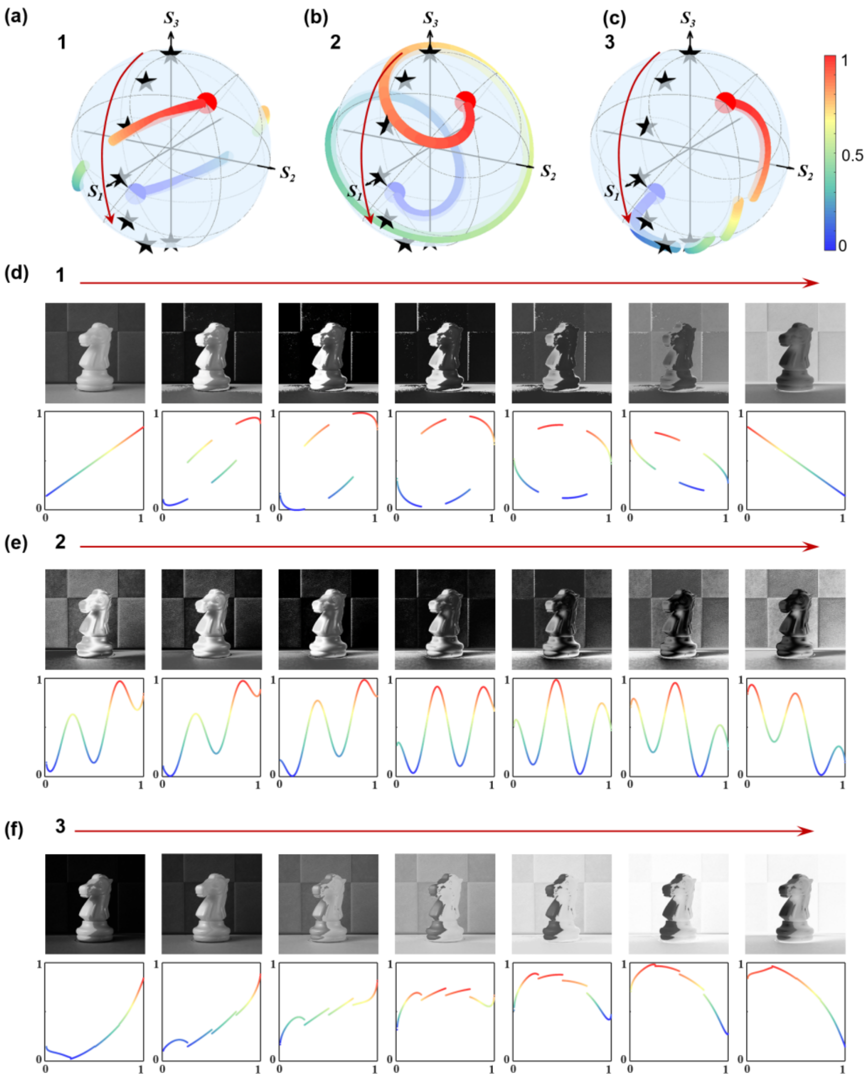

Supplementary Fig. 5. Evolution of decoded images by moving the decoding analyzer along a continuous path with fixed ψ and varying χ on the PS. (a-c) the PS modulation trajectories with functions (a) 1. $\psi_l = f(\chi_l) = \{-\pi/4$ (for $-\pi/12 \leq \chi_l < 0$ & $\pi/12 \leq \chi_l \leq \pi/4$); $\pi/4$ (for $-\pi/4 \leq \chi_l < -\pi/12$& $0 \leq \chi_l < \pi/12$)}; (b) 2. $\psi_l = 2\pi\cos^2(\chi_l - \pi/4)$ (c) 3. $\psi_l = \{5^{\cos2(\chi_l - \pi/4)+1} - 7\pi/36$ (for $-\pi/4 \leq \chi_l < -\pi/12$); π (for $-\pi/12 \leq \chi_l < 0$); $35\pi/36$ (for $0 \leq \chi_l < \pi/12$); $6^{\cos2(\chi_l - \pi/4)+1} + \pi$ (for $\pi/12 \leq \chi_l \leq \pi/4$) represented by the color-varying curves; polarization basis vectors ($|\alpha_0(50^o, 50^o)>$, $|\alpha_0^{\perp}(230^o, -50^o)>$, denoted by the red/blue dots; and decoding paths denoted by the black stars. (d, e, f) Decoded images and corresponding histogram transfer curves at the decoding paths for different modulation trajectories 1, 2, 3, respectively.

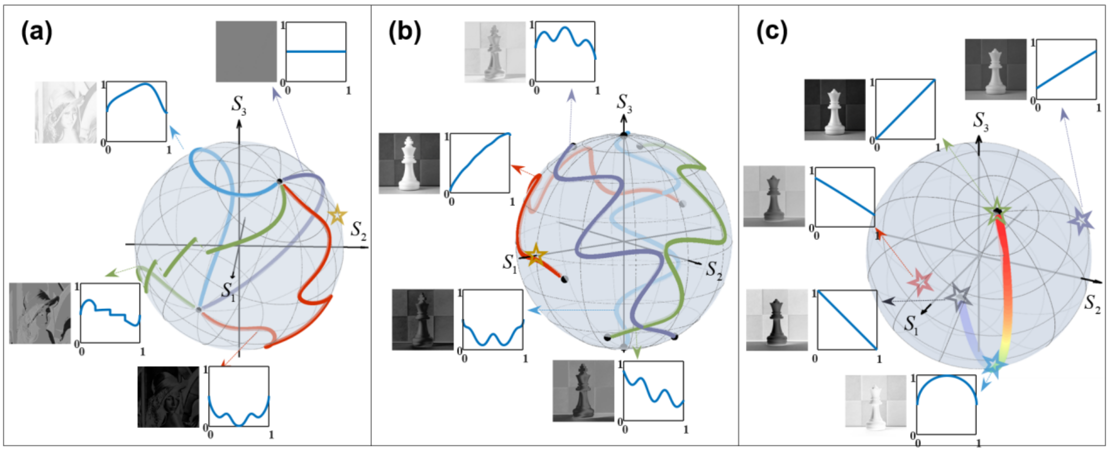

Supplementary Fig. 6. PS trajectory encoding/decoding with varying modulation trajectory, polarization basis poles and analyzer positions. (a) Encoding the spatially varying polarization information based on the same start and end points (local north/south poles) determined by $\psi_0=20^\circ$, $\chi_0=25^\circ$ (black dots), but varied modulation paths governed by different modulation functions, red: $\psi_l= 10\sin[5\pi\cos^2(\chi_l-\pi/4)]+ \pi /18$; green: $\psi_l=5^{\cos2(\chi_l -\pi/4)+1}-5\pi/12$ ($-\pi/4 \leq \chi_l < -\pi/12$); $\psi_l=7\pi/9$ ($-\pi/12 \leq \chi_l < 0$); $\psi_l=3\pi/4$ ($0 \leq \chi_l < \pi/12$); $\psi_l=6^{\cos2(\chi_l -\pi/4)+1}+7\pi/9$ ($\pi/12 \leq \chi_l \leq \pi/4$), blue: $\psi_l=[1.35\pi\cos^2(\chi_l-\pi/4)]^3+7\pi/18$, purple: $\psi_l= 5\pi/18$. Decoding analyzer at the same position by $\psi_A=117^\circ$, $\chi_A=20^\circ$ (indicated by the yellow star) yields different output polarization images and histogram transformation curves for different paths. (b) Encoding the spatially varying polarization information on the same modulation function: $\psi_l= 10\sin[5\pi\cos^2(\chi_l-\pi/4)]+2\pi/3$, but different north/south pole pairs determined by red: $\psi_0=10^\circ$, $\chi_0=-5^\circ$, green: $\psi_0=110^\circ$, $\chi_0=25^\circ$, blue: $\psi_0=170^\circ$, $\chi_0=45^\circ$, purple: $\psi_0=55^\circ$, $\chi_0=-28^\circ$. Decoding at the same analyzer points $\psi_A=0^\circ$, $\chi_A= 0^\circ$ (indicated by the yellow star) also yield different output polarization images and histogram transformation curves. (c) Encoding the polarization image with the same north/south poles and modulation trajectory, decoding analyzer at different locations on the PS (red star: $\psi_A=20^\circ$, $\chi_A= 25^\circ$, green star: $\psi_A= 142^\circ$, $\chi_A= -15^\circ$, blue star: $\psi_A= 20^\circ$, $\chi_A= -20^\circ$, purple star: $\psi_A=50^\circ$, $\chi_A= 15^\circ$) yield different output images and histogram transformations.

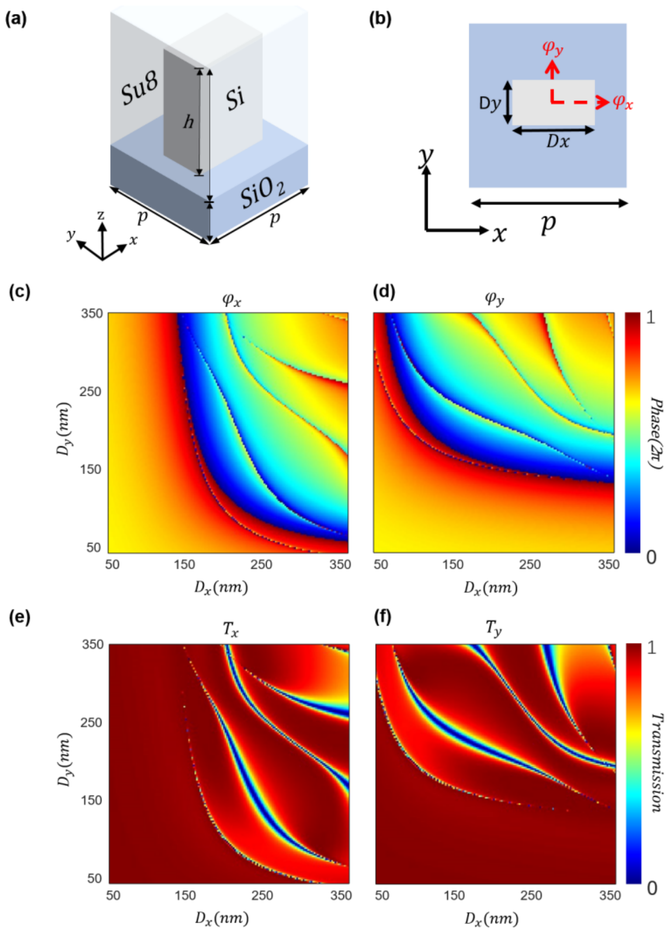

Supplementary Fig. 7. Meta-atom parameter library design for the PS trajectory encoding metasurfaces. (a) Schematic of a unit-cell of the anisotropic all-dielectric metasurface composed of a silica substrate, an amorphous silicon nanobars and an SU8 coating layer. The periodicity p=500nm, height of the meta-atom h=670nm. (b) Front view of the metasurface unit cell, the length D_x, and width D_y are varied to modulate the phase retardations φ_x, and φ_y along x-, and y-directions, respectively. (c, d) Simulated transmittance of (c) x- and (d) y-polarized incident light T_x and T_y with varying length D_x and width D_y. (e, f) Simulated phase retardation of (c) x- and (d) y-polarized incident light retardations φ_x and φ_y with varying length D_x and width D_y.

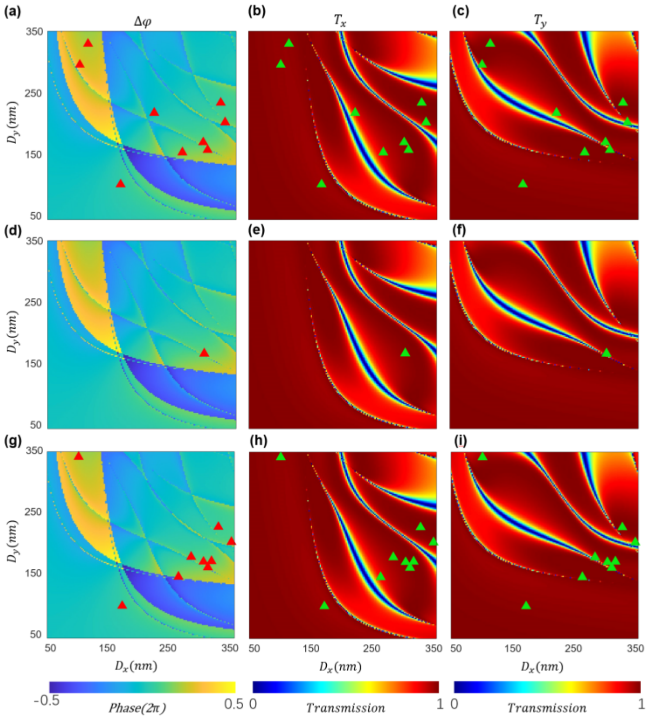

Supplementary Fig. 8. Determination of the meta-atom size parameters in the practical realized PS trajectory encoding metasurfaces. The designed D_x and D_y parameters for the experimental realized metasurfaces are denoted as triangles in the Phase retardation ($\Delta\varphi=(\varphi_x-\varphi_y)/2$) maps (a, d, g) , and Transmission efficiencies T_x (b, e, h), and T_y (c, f, i) maps of the realized metasurface with 1. $\psi_l = 2\pi\cos^2(\chi_l-\pi/4)$ trajectory under circular polarization base (a, b, c); 2. $\psi_l = f(\chi_l)=\{-\pi/4$ (for $-\pi/4 \leq \chi_l < 0$); $\pi/4$ (for $0\leq \chi_l \leq \pi/4$)$\}$ trajectory under linear polarization base (d, e, f); 3. Double local PS encoding of dual images (g, h, i).

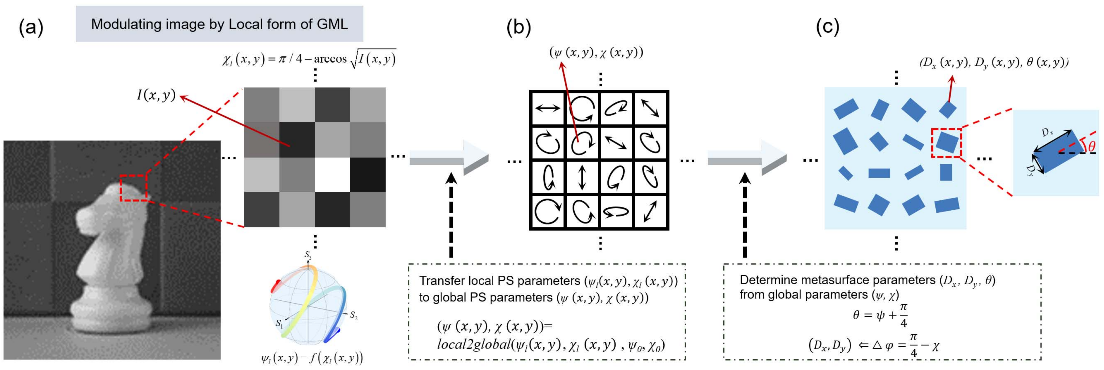

Supplementary Fig. 9. **The flowchart of the polarization information encoding with an analytic modulation trajectory.** (a) The modulated image is mapped on the local ellipticity parameter $\chi_l\ (x, y)$ based on the local form of GML, while the local azimuth parameter is encoded by an analytic function $\psi_l\ (x, y)$=f ($\chi_l\ (x, y)$). (b) Then, the local PS parameters (ψ_l, χ_l) are transformed to global PS parameters (ψ, χ) with the information of polarization basis parameters (ψ_0, χ_0). (c) After that, the global parameters (ψ, χ) are mapped to the meta-atom parameters for experimental realization.

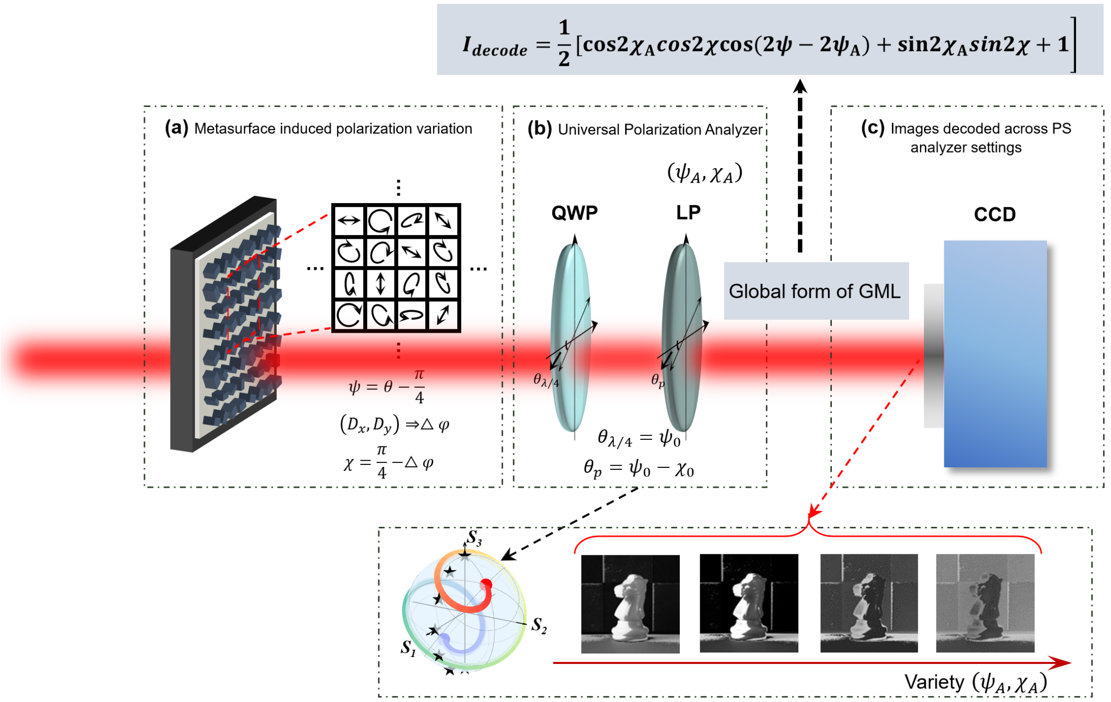

Supplementary Fig. 10. The flowchart of the decoding process with an analytic modulation trajectory. (a) In the decoder, illuminating the metasurface with circularly polarized incident light, a spatially varying profile of polarization states appears in the transmitted light beam. (b) Placing a PS universal analyzer with allowed polarization state (ψ_A, χ_A) on the transmitted light beam, the decoded intensity can be obtained by the global form of GML (c). Varying the analyzer state on the PS, the decoded intensity information will be different based on the modulated PS trajectories.

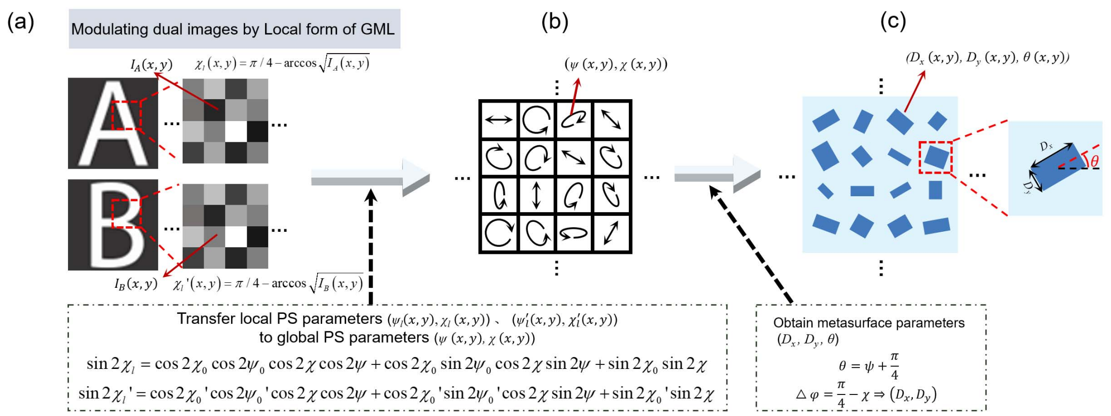

Supplementary Fig. 11. The flowchart of the polarization information encoding with double information channel encryption. (a) In the encoder, two sets of polarization images 'A' and 'B' are mapped on two local PS systems with ellipticity parameter $\chi_l(x, y)$ and $\chi_l'(x, y)$, respectively. (b) Then, the two sets of local ellipticity $\chi_l(x, y)$ and $\chi_l'(x, y)$ are transformed to one sets of PS parameters (ψ, χ) with the information of polarization basis parameters (ψ_0, χ_0) and (ψ_0', χ_0') (c). After that, the global parameters (ψ, χ) are mapped to the meta-atom parameters for experimental realization.

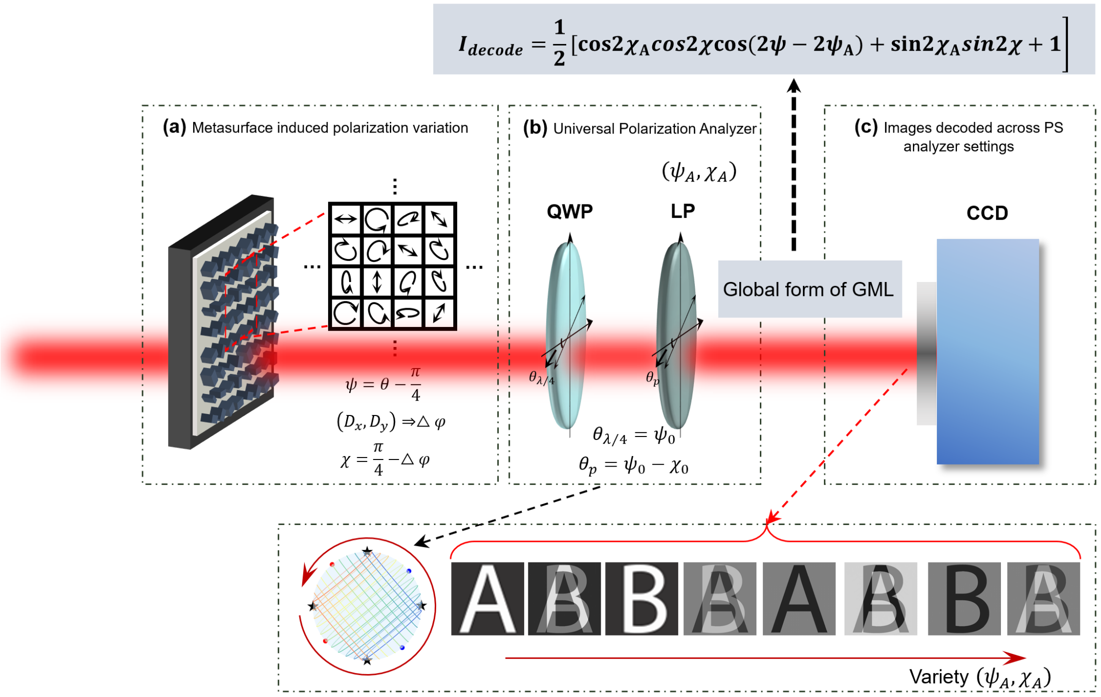

Supplementary Fig. 12. The flowchart of the decoding process with double information channel encryption. (a) In the decoder, illuminating the metasurface with circularly polarized incident light, a spatially varying profile of polarization states appears in the transmitted light beam. (b) Placing a PS universal analyzer with allowed polarization state (ψ_A, χ_A) on the transmitted light

beam, the decoded intensity can be obtained by the global form of GML (c). Varying the analyzer state along a circular path on the PS, the decoded images emerge as original, grayscale-reversed and mixed versions of ‘A’ and ‘B’ images at particular analyzer locations on the PS.

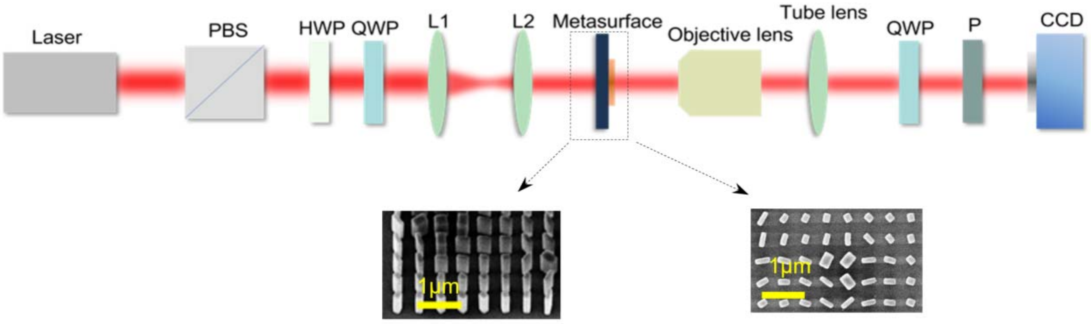

Supplementary Fig. 13. Experimental setup for the metasurface characterization. PBS: polarization beam splitter, HWP: half waveplate, QWP: quarter waveplate, L1, L2: lens, P: polarizer. Bottom panels show the SEM image of tilted- and front-view of a small range of the fabricated metasurfaces.

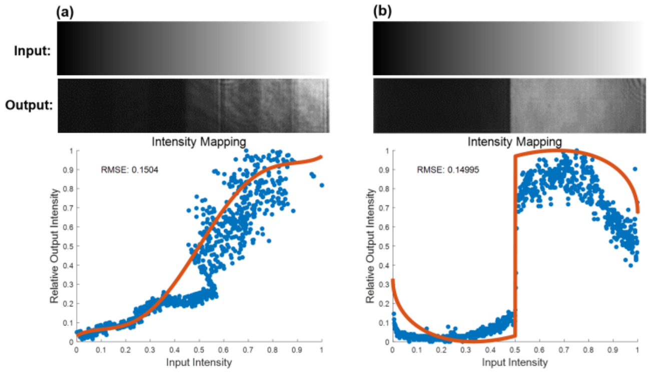

Supplementary Fig. 14. Linearly gradient grayscale images (upper panels) and their input-output relations (lower panels) modulated by (a) the *S*-shape and (b) the thresholding curves. We sample points on the captured output images, and plot the relation between the experimental captured intensity and that of the original image. For both the *S*-shape and thresholding curve modulation, the experimental sampled points gather towards a certain area near the theoretical input-output transfer curve. The root-mean square error (RMSE) defined as $RMSE = \sqrt{\frac{\sum_{i=1}^{n}(I_{experiment,i}-I_{theory,i})^2}{n}}$ (where $I_{experiment,i}$ and $I_{theory,i}$ are the experimental measured and the corresponding theoretic value of the output intensity, n is the total number sampled points) is used to quantify the discrepancy between the experimental data and the theoretical value. The RMSEs for the experimental data of the S-shape and thresholding curve are 0.15040, 0.14995, respectively, showing relative accuracy of the reconstructed images.

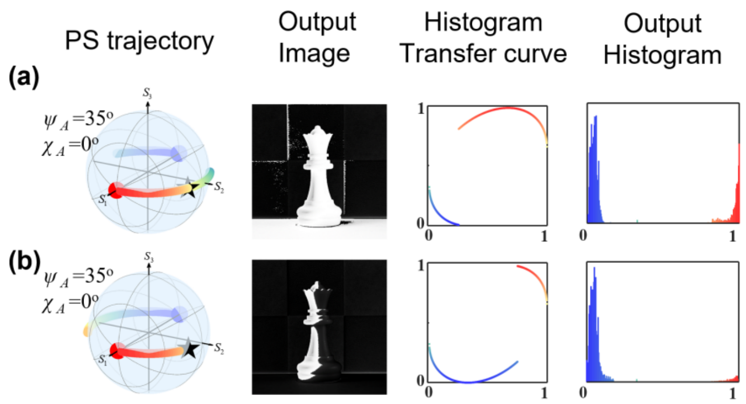

Supplementary Fig. 15. Designed thresholding PS trajectory encoding with a threshold value (a) 0.25 and (b) 0.75. The modulation function is (a) $\psi_l = f(\chi_l)$= -π/4 (-π/4 ≤ χ_l <-π/12), π/4 (-π/12 ≤ χ_l ≤π/4); (b) $\psi_l = f(\chi_l)$= -π/4 (-π/4 ≤ χ_l < π/12), π/4 (π/12 ≤ χ_l ≤π/4) , respectively. The decoded images, histogram transfer function, and output histograms are obtained by setting the decoding analyzer on the PS at ψ_A=35°, χ_A=0°.

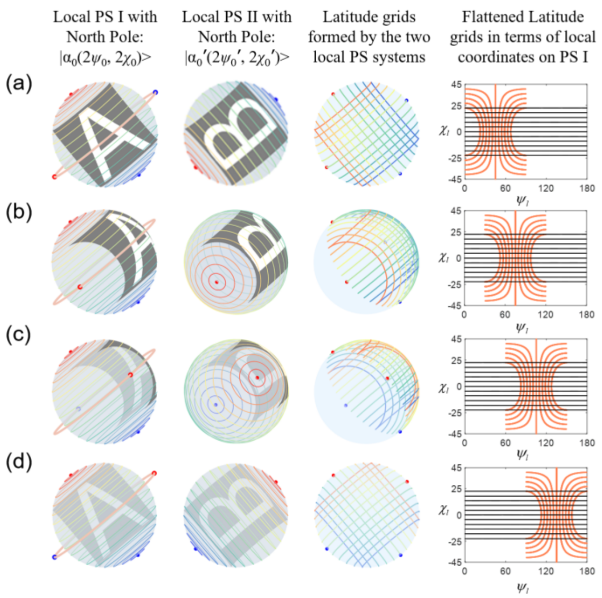

Supplementary Fig. 16. The general design strategy for PS grid encoding of dual images by arbitrarily aligned two polarization bases. Encoding Image 'A' on a local PS (PS I) with polarization basis poles (|$\alpha_0(2\psi_0, 2\chi_0)$>, |$\alpha_0^{\perp}(2\psi_0+\pi, -2\chi_0)$>) (denoted as the red and blue dots, left-most panels); Another image 'B' could be independently encoded by another local PS (PS II) with polarization basis poles (|$\alpha_0'(2\psi_0', 2\chi_0')$>, |$\alpha_0'^{\perp}(2\psi_0'+\pi, -2\chi_0')$>) (second column panels). (a-d) By setting the polarization basis poles of PS II on the equator of the local PS I, perpendicular latitude grids can be produced for independently modulating the dual images, with explicit modulation expressions (Eqs. 62). Once the local PS I is set, there are infinite number of choices of PS II for such perpendicular grid, as long as its north/south poles are along the circle defined by the equator of PS I. In this way, the modulation region defined by those two sets of local latitudes on the PS rotate correspondingly (the 3rd column panels). The rotating of the modulation grid area can be intuitively illustrated on the local coordinate (ψ_l, χ_l) of PS I, as shown in the right-most panels, where the black lines are latitudes of local PS I, while the red curves are latitudes of local PS II. The area of their interaction points represents the modulation parameters range in terms of local coordinates of PS I.

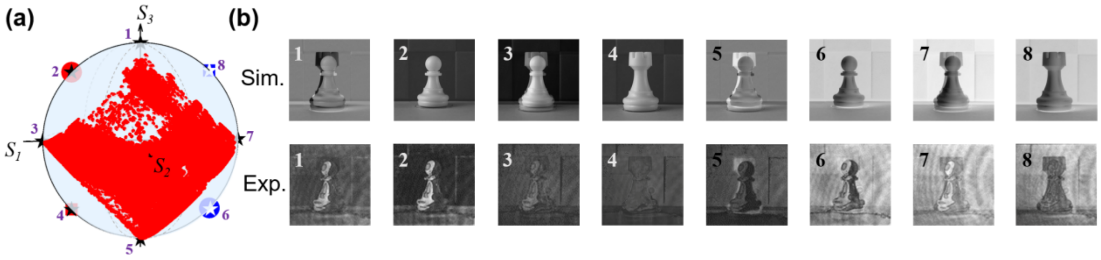

Supplementary Fig. 17. Experimental realization of metasurface V for PS trajectory encoding with two non-orthogonal elliptical polarization state bases as the dual information channels. (a) Experimental realization of dual information channel encoding by setting PS I on the elliptical basis (ψ_I=0° ,χ_I =22.5°) and PS II on the other elliptical basis (ψ_I=0° ,χ_I =-22.5°). (b) The simulated and experimental decoding images at positions 1-8 surrounding the modulation grids. The images of 'pawn' and 'rook' are decoded corresponding to the elliptical polarization 2 (ψ_A=0°, χ_A=22.5°) and elliptical polarization 4 (ψ_A=0° ,χ_A=-22.5°) states, which correspond to the polarization base north pole of PS I and PS II, respectively. The intermediary positions 1 (ψ_A=0°, χ_A=45°), 3 (ψ_A=0°, χ_A=0°), 5 (ψ_A=0°, χ_A=-45°) ,7 (ψ_A=90°, χ_A=0°) result in the generation of mixed images. Notably, the images appearing at polarization states 6 (ψ_A=90°, χ_A=-22.5°) and 8 (ψ_A=0°, χ_A=22.5°) exhibit grayscale inversed versions of the images at polarization states 2 and 4, respectively.

References

1. Chipman, R. A., Lam, W.-S. T. & Young, G. *Polarized light and optical systems*. (CRC press, 2018).
2. Collett, E. & Schaefer, B. Visualization and calculation of polarized light. I. The polarization ellipse, the Poincaré sphere and the hybrid polarization sphere. *Appl. Opt.* **47**, 4009-4016 (2008).
3. Milione, G., Sztul, H. I., Nolan, D. A. & Alfano, R. R. Higher-Order Poincaré Sphere, Stokes Parameters, and the Angular Momentum of Light. *Phys. Rev. Lett.* **107**, 053601 (2011).
4. Naidoo, D.*, et al.* Controlled generation of higher-order Poincaré sphere beams from a laser. *Nat. Photon.* **10**, 327-332 (2016).
5. Liu, M.*, et al.* Broadband generation of perfect Poincaré beams via dielectric spin-multiplexed metasurface. *Nat. Commun.* **12**, 2230 (2021).
6. Fu, S., Wang, T., Zhang, Z., Zhai, Y. & Gao, C. Selective acquisition of multiple states on hybrid Poincare sphere. *Appl. Phys. Lett.* **110**, 191102 (2017).
7. Voitiv, A. A., Lusk, M. T. & Siemens, M. E. Tilted Poincaré sphere geodesics. *Opt. Lett.* **47**, 1089-1092 (2022).
8. Song, Q., Odeh, M., Zúñiga-Pérez, J., Kanté, B. & Genevet, P. Plasmonic topological metasurface by encircling an exceptional point. *Science* **373**, 1133-1137 (2021).
9. Wang, S.*, et al.* Arbitrary polarization conversion dichroism metasurfaces for all-in-one full Poincaré sphere polarizers. *Light Sci. & Appl.* **10**, 24 (2021).
10. Overvig, A., Yu, N. & Alù, A. Chiral Quasi-Bound States in the Continuum. *Phys. Rev. Lett.* **126**, 073001 (2021).
11. Menzel, C., Rockstuhl, C. & Lederer, F. Advanced Jones calculus for the classification of periodic metamaterials. *Phys. Rev. A* **82**, 053811 (2010).
12. Pfeiffer, C., Zhang, C., Ray, V., Guo, L. J. & Grbic, A. High Performance Bianisotropic Metasurfaces: Asymmetric Transmission of Light. *Phys. Rev. Lett.* **113**, 023902 (2014).
13. Song, Q.*, et al.* Ptychography retrieval of fully polarized holograms from geometric-phase metasurfaces. *Nat. Commun.* **11**, 2651 (2020).
14. Wang, S., Wen, S., Deng, Z. L., Li, X. & Yang, Y. Metasurface-Based Solid Poincare Sphere Polarizer. *Phys Rev Lett* **130**, 123801 (2023).
15. Huang, C.-p., Wang, Y.-l. & Zhang, Y. Interference-type plasmonic polarizers and generalized law of Malus. *J. Opt.* **21**, 105001 (2019).
16. Arbabi, A., Horie, Y., Bagheri, M. & Faraon, A. Dielectric metasurfaces for complete control of phase and polarization with subwavelength spatial resolution and high transmission. *Nat. Nanotechnol.* **10**, 937-943 (2015).
17. Balthasar Mueller, J. P., Rubin, N. A., Devlin, R. C., Groever, B. & Capasso, F. Metasurface Polarization Optics: Independent Phase Control of Arbitrary Orthogonal States of Polarization. *Phys. Rev. Lett.* **118**, 113901 (2017).
18. Devlin, R. C., Ambrosio, A., Rubin, N. A., Mueller, J. P. B. & Capasso, F. Arbitrary spin-to-orbital angular momentum conversion of light. *Science* **358**, 896–901 (2017).
19. Guo, X.*, et al.* Tying Polarization-Switchable Optical Vortex Knots and Links via Holographic All-Dielectric Metasurfaces. *Laser Photon. Rev.* **14**, 1900366 (2020).
20. Bao, Y., Ni, J. & Qiu, C.-W. A Minimalist Single-Layer Metasurface for Arbitrary and Full Control of Vector Vortex Beams. *Adv. Mater.* **32**, 1905659 (2020).

21. Lung, S., *et al.* Complex-Birefringent Dielectric Metasurfaces for Arbitrary Polarization-Pair Transformations. *ACS Photon.* **7**, 3015-3022 (2020).
22. Khorasaninejad, M., Zhu, W. & Crozier, K. B. Efficient polarization beam splitter pixels based on a dielectric metasurface. *Optica* **2**, 376 (2015).
23. Yu, Y. F., *et al.* High-transmission dielectric metasurface with 2π phase control at visible wavelengths. *Laser Photon. Rev.* **9**, 412-418 (2015).
24. Genevet, P., Capasso, F., Aieta, F., Khorasaninejad, M. & Devlin, R. Recent advances in planar optics: from plasmonic to dielectric metasurfaces. *Optica* **4**, 139-152 (2017).
25. Arbabi, E., Kamali, S. M., Arbabi, A. & Faraon, A. Vectorial Holograms with a Dielectric Metasurface: Ultimate Polarization Pattern Generation. *ACS Photon.* **6**, 2712-2718 (2019).
26. Song, Q., *et al.* Bandwidth-unlimited polarization-maintaining metasurfaces. *Sci. Adv.* **7**, eabe1112 (2021).
27. Yu, N., *et al.* A Broadband, Background-Free Quarter-Wave Plate Based on Plasmonic Metasurfaces. *Nano Lett.* **12**, 6328-6333 (2012).
