# Supplementary material for: Poincaré sphere trajectory encoding metasurfaces based on generalized Malus’ law
Source: Nat Commun. 2024 Mar 16;15:2380. doi: 10.1038/s41467-024-46758-y (PMC10944530; doi:10.1038/s41467-024-46758-y)
Supplement: Supplementary file 3 — Description of Additional Supplementary Files [file 41467_2024_46758_MOESM3_ESM.pdf]

Description of Additional Supplementary Files

File Name: Supplementary Movie 1

Description: Poincare sphere trajectory encoding/decoding with varying decoding analyser states.

File Name: Supplementary Movie 2

Description: Poincare sphere trajectory encoding/decoding with varying modulation trajectories.

File Name: Supplementary Movie 3

Description: Poincare sphere trajectory encoding/decoding with varying encoding polarization basis.

File Name: Supplementary Movie 4

Description: Modulating PSII along the equator of PSI for advanced PS trajectory encoding with a nonorthogonal aligned modulation grid for dual information channels.
